# High Costs of Female Choice in a Lekking Lizard

**DOI:** 10.1371/journal.pone.0000567

**Published:** 2007-06-27

**Authors:** Maren N. Vitousek, Mark A. Mitchell, Anthony J. Woakes, Michael D. Niemack, Martin Wikelski

**Affiliations:** 1 Department of Ecology and Evolutionary Biology, Princeton University, Princeton, New Jersey, United States of America; 2 Department of Veterinary Clinical Sciences, Louisiana State University, Baton Rouge, Louisiana, United States of America; 3 Center for Ornithology, School of Biosciences, University of Birmingham, Edgbaston, United Kingdom; 4 Department of Physics, Princeton University, Princeton, New Jersey, United States of America; University of Exeter, Cornwall Campus, United Kingdom

## Abstract

Although the cost of mate choice is an essential component of the evolution and maintenance of sexual selection, the energetic cost of female choice has not previously been assessed directly. Here we report that females can incur high energetic costs as a result of discriminating among potential mates. We used heart rate biologging to quantify energetic expenditure in lek-mating female Galápagos marine iguanas *(Amblyrhynchus cristatus)*. Receptive females spent 78.9±23.2 kJ of energy on mate choice over a 30-day period, which is equivalent to ∼¾ of one day's energy budget. Females that spent more time on the territories of high-quality, high-activity males displayed greater energetic expenditure on mate choice, lost more mass, and showed a trend towards producing smaller follicles. Choosy females also appear to face a reduced probability of survival if El Niño conditions occur in the year following breeding. These findings indicate that female choice can carry significant costs, and suggest that the benefits that lek-mating females gain through mating with a preferred male may be higher than previously predicted.

## Introduction

Optimal mate choice involves a tradeoff between the cost of selecting a mate and the benefit gained by mating with a preferred partner. Substantial research has centered on identifying the particular benefits females accrue through discriminating among potential mates, but very little is known about the corresponding costs of choice or their magnitude. Recent studies indicate that a fitness cost can be associated with mate choice, and that females alter their selectivity when costs differ [1]–[5]. The specific nature of mate choice costs and their relationship to increasing mate sampling effort remains unclear.

Increasing evidence suggests that mate choice can be maintained by indirect genetic benefits (the production of genetically superior offspring) in a variety of species [6]–[9]; however, the magnitude of genetic benefits is generally considered to be small [10], [11]. If this is the case, indirect benefits could only maintain mate choice if the costs of choosing are low. Despite the plethora of studies on the genetic benefits of mate choice, the cost of choosing a mate has not previously been directly quantified in any species.

Sexual selection and male mating skew are unusually strong in lekking species [12], [13], even though lek-mating females typically receive no direct benefits (e.g. food resources or parental care) from their chosen mate. This phenomenon has been termed the ‘paradox of the lek’ [10], [14]. It has often been assumed that the assessment of lekking males incurs low energetic costs because of the tight clustering of territories [10], [15]; however, there is little empirical support for this theory. A resolution of the lek paradox depends on determining the cost of female choice.

Two studies have indirectly estimated the energetic cost of mate choice by calculating the energetic expenditure predicted to result from the increased distance females travel while assessing males. In female sage grouse *(Centrocercus urophasianus)*, traveling to leks was estimated to represent ∼1% of one day's energy expenditure, and to lower annual survival by ∼0.1% [16]. Any costs incurred while on the lek were not included, and birds were not observed off the lek. Female choice over a two-week period was estimated to increase the energetic expenditure of female pronghorn antelope *(Antilocapra americana)* by ∼50% of one day's energy budget [17]. While these studies provide useful estimations of the energetic cost of choice, calculations based on the distance traveled provide an inevitably imprecise estimate of cost. Additionally, the relationship between energetic expenditure on female choice and resulting physiological or fitness costs was not investigated in these species.

We quantified the energetic cost of mate choice in the Galápagos marine iguana *(Amblyrhynchus cristatus)*, a lekking species in which the largest males defend display territories during the mating season that do not contain any energetic rewards. Females visit these territories over a period of approximately 45 days, and maintain nearly constant presence on the territories during this interval. Territorial males display to visiting females using a stereotyped side-walking head-bob display. Resident males court visiting females almost continuously throughout the mate choice period, but males within a single lek can differ 10-fold in courtship display rate. Energy expenditure on locomotion represents a significant portion of the energy budget of iguanids [18], and territorial males fast for the duration of the mate choice period, thus the ability to maintain costly courtship displays may be an honest indicator of male quality.

Females preferentially visit territories containing larger males, and among the territories that they visit, typically mate with the male that is most active during the time they are observing [19]. The chosen mate is not always the male that displays the most overall, indicating that females make independent decisions based on the information that they gather. Visiting the territories of active males may be costly to female marine iguanas: females reject courtship and copulation attempts by moving several steps away from the displaying male.

The timing of mating is relatively synchronous within a population, occurring over a period of approximately 14 days. Territorial males do not force copulations or attempt to keep females from leaving territories; thus females can move freely between territories [20], [21]. Females do not copulate preferentially at territories that received high copulation rates in previous years [22], and cluster locations are unrelated to habitat characteristics or non-breeding female densities [21]. Receptive females normally mate only once per reproductive season [23]. Females were classified as receptive if they were observed copulating with a territorial male or had developing follicles when assessed by ultrasound at the conclusion of the mate choice period. Marine iguanas typically mate biennially, which allows comparison of the costs incurred by receptive and non-receptive females of the same size and weight classes within a season.

The mass-specific oxygen pulse of *A. cristatus*, defined as the product of cardiac stroke volume and the difference in oxygenation between arterial and mixed venous blood, varies predictably with exercise [24]. This makes it possible to determine energetic expenditure on a fine temporal scale from recorded values of heart rate and body temperature. We correlated these fine-scale measurements of metabolic rate with behavioral observations taken continuously throughout the mating season.

## Results

Receptive female marine iguanas spent a mean of 78.9 (±23.2) kJ of energy on mate choice behaviors during the 30 days prior to mating, which is equivalent to 78.4% of one day's energetic expenditure during the reproductive season (100.6±16.8 kJ/day). During most years all marine iguanas are in negative energy balance during the reproductive period due to seasonal fluctuations in algal abundance; however, receptive females tended to lose more mass during the mate choice period than non-receptive females (receptive: 13.6±1.6%, *n* = 18; non-receptive: 7.6±2.3, *n* = 7; *t* = −2.06, *df* = 23, *P* = 0.051; *r* = 0.425). Females that lost more mass also showed a trend towards spending more energy on mate choice (*r* = 0.523, *n* = 13, *P* = 0.066). Receptive and non-receptive females did not differ in their initial body mass (receptive: 0.993±0.153, *n* = 18; non-receptive: 0.932±0.151, *n* = 7; *t* = −0.901, *df* = 23, *P* = 0.377; *r* = 0.197) or snout-vent length (receptive: 25.1±1.5, *n* = 16; non-receptive: 25.6±1.5, *n* = 7; *t* = 0.629, *df* = 21, *P* = 0.536; *r* = −0.164).

**Figure 1:**
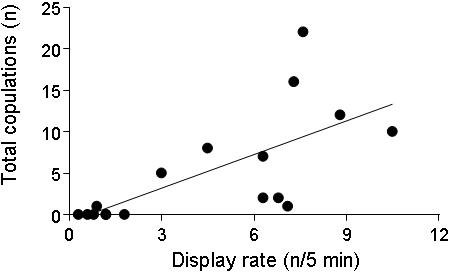
Male display rate and copulation success. Male display rate, measured as mean number of courtship displays per 5-minute period, significantly predicted copulation success (ANOVA; F*_1,13_* = 10.704, *P* = 0.006).

Male territorial behavior significantly predicted copulation success (ANOVA: *F_3,13_* = 5.452, *P* = 0.012, *r* ^2^ = 0.557). Specifically, the number of copulations a male received was significantly associated with his rate of head-bobbing display (Fig 1; *F_1,13_* = 10.704, *P* = 0.006), but not with the frequency of his participation in territorial fights (*F_1,13_* = 2.456, *P* = 0.141) or chases (*F_1,13_* = 0.123, *P* = 0.731). Males were assigned quality scores (1–5) based on copulation success, and the mean quality of males that each female visited was calculated, weighted for time spent with each male.

**Figure 2:**
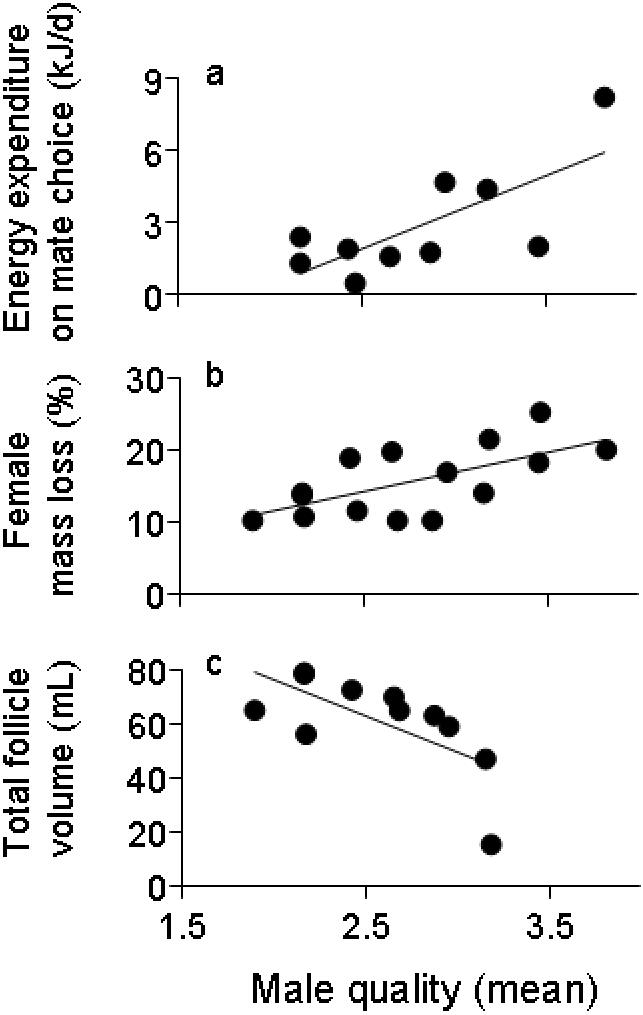
Relationships between mean male quality and female mate choice, mass loss and follicle size. Female marine iguanas that spent more time on the territories of high-quality, high activity males (a) spent more energy on mate choice (*r* = 0.731, *n* = 10, *P* = 0.016), (b) lost more body mass during the mate choice period (*r* = 0.646, *n* = 15, *P* = 0.009), and (c) displayed a trend towards producing smaller follicles (*r* = −0.660, *n* = 11, *P* = 0.027). Males were assigned quality scores (1–5) based on copulation success. Data represent the mean quality score of the males a female visited, weighted for time spent with each male.

Relationships among mean male quality, follicular volume, mass, and mate choice are listed in Table 1. Among receptive females there was no relationship between initial body mass and the quality of males visited; however, receptive females that visited high-quality males spent more energy on mate choice, lost more mass, and showed a strong trend towards developing smaller follicles (Fig 2a–c). Females that visited high-quality males did not spend more time on territories overall. Follicular volume was not correlated with initial body mass, or with mass loss during mate choice, but females that mated later in the season developed larger follicles.

**Table 1:** *P*-values that are significant after correction using false discovery rate are indicated by *.

Variables	*r*	*n*	*P*
Male quality vs. initial body mass	0.358	15	0.191
Male quality vs. % mass loss	0.646	15	0.009*
Male quality vs. EE on mate choice	0.731	10	0.016*
Male quality vs. % time on territories	0.301	7	0.512
Male quality vs. follicle volume	−0.660	11	0.027
Follicle volume vs. initial mass	−0.102	12	0.752
Follicle volume vs. % mass loss	−0.250	12	0.434
Follicle volume vs. mating date	0.860	9	0.003*

While stationary on territories, receptive females spent a higher proportion of time sitting (head and upper body elevated on front limbs) than non-receptive females (receptive: sit:lay 2.06±0.14, *n* = 11, non-receptive: 1.45±0.16, *n* = 4; *t* = −2.42, *df* = 13, *P* = 0.031; *r* = 0.609). Energetic expenditure per hour was higher while in the sitting posture than while laying (sitting: 3.33±0.85 kJ*hr^−1^, laying: 2.76±0.79; paired *t*-test, *t* = −4.28, *n* = 9, *P* = 0.003; *r* = 0.115).

## Discussion

Mate choice represents a relatively high cost to female marine iguanas; receptive females spent a mean of 78.9 (±23.2) kJ of energy on mate choice over a 30-day period, which is equivalent to 78% of one day's energy budget. The relative energetic expenditure on female choice in marine iguanas is 1.5 times what was estimated in pronghorn antelope [17] and over 50 times the cost of traveling to leks in female sage grouse [16].

Our results suggest that assessing high-quality males is more costly than assessing low-quality males. Receptive females that spent more time visiting high-quality males displayed greater energy expenditure on mate choice and lost more body mass during the mate choice period. Although the correlational design of our study does not enable us to determine whether these variables are causally related, we were unable to conduct controlled experiments for the following reasons: 1) female mate choice cannot be experimentally manipulated in free-roaming marine iguanas; 2) captive animals have very low survival rates; and 3) marine iguanas do not exhibit mate choice or mating behaviors in enclosure experiments, even when these enclosures are located on the territories. Although we cannot conclusively assess whether the increased mass loss suffered by ‘choosy’ females is a direct result of the increased time spent with high-quality males, we suggest that this is the most likely explanation. Females that spent more time with high-quality males did not have higher initial body masses, or differ in the total amount of time spent visiting territories. High-quality males (males with high copulation rates) maintain nearly constant territorial and courtship displays during the mating season. These displays typically induce visiting females to move a few steps away from the displaying male and incur the resulting measurable energetic cost. As predicted, females that spent more time with high-quality males spent more energy on mate choice behaviors (which include these movements away from displaying males). Thus we suggest that the energetic cost incurred by moving away from the frequent displays of high-quality males contributes to the parallel loss of body mass observed in females visiting these males.

Marine iguanas are in the process of growing follicles during the mate choice period, an activity which represents a significant energetic cost to females [20], [25], and likely contributes to the high degree of mass loss observed in receptive females. There may also be additional costs associated with male assessment that were not included in our definition of mate choice behaviors. Receptive females spent significantly less time lying down, tending instead to adopt the more energetically costly sitting posture while stationary on territories. The elevation of the head and upper body that is characteristic of the sitting posture likely enables these females to maintain continual observation of their surroundings, including the location and behavior of territorial males. Increased opportunities to monitor potential mates may provide receptive females with benefits that balance the additional costs of remaining in the sitting posture. Additionally, our estimations of energetic expenditure on mate choice are conservative: in calculating the energy spent on a mate choice event we included elevations in energetic expenditure during the encounter, and for up to three minutes following the cessation of locomotion. Some studies suggest that reptiles may exhibit increases in energetic expenditure for a longer duration (>10 mins) following the termination of activity [26]; thus it is possible that our conservative estimate of mate choice underestimates the proportion of total energetic expenditure due to mate choice and other locomotory events.

The mass loss resulting from mate choice behavior is a biologically significant cost to marine iguanas due to the frequent and unpredictable occurrence of El Niño conditions in the Galápagos. During El Niño years an increase in sea surface temperature causes a decline in the algal food resources utilized by marine iguanas, and can result in massive mortality. Animals with low body weight at the start of an El Niño year have a dramatically lower probability of surviving the season [27]. Females that breed in the year prior to an El Niño face higher mortality rates during El Niño than non-breeding females, indicating that reproductive effort can have serious fitness consequences [27]. Females that spend more energy on mate choice, and thus lose more mass, should face a substantially diminished probability of survival if El Niño conditions prevail during the year following reproduction. Based on previous survival estimates in this population [28], we predict that the increased mass loss suffered by choosy females (8.1% of body mass) would result in only a marginal (0–2%) decrease in annual survival if a cool La Niña year follows reproduction. If a choosy female is subjected to El Niño conditions after mating, however, her survival probability may decrease by as much as 14% compared to a non-choosy female.

The energetic cost of mate choice in marine iguana females is also relevant to reproduction because individuals appear to face a trade-off between allocating resources to mate choice and to follicular development. Although follicle volume was not related to a female's initial body mass or mass loss, females that visited high-quality males showed a strong trend towards producing smaller follicles. Follicle size is positively correlated with hatchling size in many lizard species [29]–[31], and hatchling size is a significant predictor of survival in marine iguanas [32], [33]. Females that visited higher-quality males also showed a tendency to mate earlier in the season. While further study is needed to explore this result, our findings do not support the alternative hypothesis that females that mate with a high-quality male take longer to assess males and develop follicles, which are thus being measured at an earlier stage of development.

The possibility that females are receiving direct benefits from high-quality males is difficult to conclusively rule out, as each potential benefit must be tested individually; however, we have found no evidence to date that females receive any direct benefits from visiting or mating with a higher-quality male. No food resources are located in territories, and there are no detectable differences in the microclimate (topography or thermal properties) of areas defended by males and other areas containing groups of iguanas [21]. Females visiting high-quality territorial males could potentially receive the benefit of greater protection from forced copulation attempts by satellite males. Satellite males are present on the periphery of territories and attempt to solicit copulations from females as they move between territories or travel to foraging areas. Females face a lower risk of forced copulation attempts on territories than off territories [21], but we found no relationship between territorial male quality and the number of territorial intrusions, indicating that beyond the benefit of being on a territory, females do not gain additional protection from visiting higher-quality males.

We were not able to conduct genetic analyses of paternity on the offspring of study females due to the difficulty of individually fencing nest sites in the high-density communal nesting grounds; however, we believe that it is reasonable to assume that in most cases the male a female is observed to mate with is the genetic father of her offspring. It is rare for females to mate more than once in a season, and in most of the small percentage of cases in which this has been observed, both copulations were with the same male.

Here we demonstrate that female choice incurs an energetic cost that appears to translate into direct costs (mass loss/decreased survival probability), and possibly also indirect costs (decreased clutch mass). We found no evidence that females gain any direct benefits through mating with a high-quality male; thus we suggest that the maintenance of costly mate choice behavior may be related to the potential genetic benefit conferred to a female's offspring [34]. Females that sample more males ultimately mate with a more active and larger male [19]. Body size appears to be highly heritable in iguanas [35], so large males are likely to pass this element of attractiveness on to their sons. Our finding of a high cost of mate choice in a lekking species in which females appear to gain no direct benefits from territorial males is contrary to theoretical predictions that only low choice costs can be maintained by indirect genetic benefits [10], [36]. Determining the cost of mate choice in a diversity of mating systems is critical to understanding the evolution and maintenance of sexual selection.

## Methods

### Location and Measurement

Data were collected at Bahia Paraiso on Isla Santa Fe, Galápagos Archipelago, Ecuador (90°2′W, 0°50′S). We obtained behavioral and morphological data from 23 females, and heart rate-body temperature data from 11 females: 7 in 2004–2005 and 2 each in 1999–2000 and 2003–2004. Marine iguanas were captured either by hand or with the aid of a loop at the end of a bamboo pole. Snout vent length (SVL) and body mass were recorded both at the time of initial capture and after recapture at the conclusion of the mate choice period. Focal females and all territorial males were identified through small numbers painted on their flanks using non-permanent paint; these markings do not affect attractiveness in marine iguanas (M. Wikelski, unpublished data).

### Data Logger Implantations

Female marine iguanas were anaesthetized using an intravenous injection of propofol at 15 mg/kg. The iguanas were intubated using a 2–0 endotracheal tube (outside diameter) and ventilated (6 times/min) using an ambu-bag. The surgical site was prepared using aseptic techniques. The sterilized heart-rate data logger was inserted into the coelomic cavity through a 4–5 cm paramedian incision through the skin and underlying muscles that initiated just caudal to the xiphoid process on the sternum and extended caudally. The data logger was secured in the coelomic cavity by tacking two simple interrupted absorbable sutures (3–0 polydioxanone; Ethicon Inc., Cornelia, GA USA) to the body wall. The body wall and skin were closed using an absorbable suture (3–0, 2–0 polydioxanone). Recovery from surgery and anesthesia was uneventful. Iguanas were held in a warm location for a minimum of two hours post-surgery, and were then released at their capture site. At the conclusion of the study period the data loggers were removed through a parallel incision using the same surgical techniques.

Loggers weigh ∼15 g, representing about 2% of female body weight. A control group of non-implanted females was maintained, subject to the same handling procedures minus the surgical process and implantation. Marine iguanas do not appear to suffer major post-surgical trauma. Corticosterone levels, assessed through a blood sample taken from the caudal vein 24 h after surgery, do not differ between implanted and control females [24]. After five days post-surgery, implanted and control females do not differ in the number of males assessed per day (implant: 2.9±0.9, *n* = 10; control: 2.9±0.8, *n* = 4; *t* = −0.14, *df* = 12, *P* = 0.891) or inter-territorial movements (implant: 4.4±2.4, *n* = 10; control: 4.1±2.2, *n* = 4; *t* = −0.19, *df* = 12, *P* = 0.857). No mortality occurred during the study, and implanted animals and controls show no difference in annual survival rates.

### Behavioral Observations

Five-minute focal behavioral samples were conducted on all territorial males every 1.5 days for the duration of territory tenure. During the five-minute observation period the number of male displays, chases, and fights were recorded. Focal behavioral samples of study females were conducted continuously throughout the mating season during all daylight hours (0600 h to 1800 h), the entirety of the active period in marine iguanas [37]. During focal observations the focal female's behavior and her interactions were recorded, as were the identity of the individuals with whom the focal female interacted, the male territory visited, and her location. Marine iguanas are highly site-faithful, and the topography of the study site allows individuals to be observed continually during mate choice, copulation, foraging, and resting.

We classified mate choice behaviors as: 1) movement between male territories (not including territories on a direct path to the foraging grounds); 2) direct approach towards a male by the focal female, which characteristically elicits male display; 3) male courtship display directed at a female that caused her to move away from the approaching male.

### Quantification of Energetic Expenditure

Heart rate (*f*H) and body temperature (T_b_) were measured with implanted data loggers that record both variables at 2 s intervals. Measurements of *f*H and T_b_ were subsequently averaged over 60 s periods. The mass-specific rate of oxygen consumption (s*V*_O2_), in units of ml*g^−1^*h^−1^, was calculated using a modified version of Fick's convection equation for the cardiovascular system adapted for use in marine iguanas [24]:
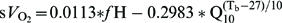
Q_10_ was set at 2.0. Oxygen consumption was converted to metabolic rate using a respiratory quotient (RQ) of 0.8 [38].

### Follicular Measurement

In 2004–2005 the size of developing follicles was measured after the last copulation on the study site using ultrasonography [39]. The presence and size of developing follicles was highly repeatable in marine iguanas, and after an initial trial period showed high inter-observer reliability (M.N. Vitousek&M.A. Mitchell, unpublished data). We were unable to obtain a measurement of follicular volume in one of the females upon recapture due to the presence of digestive matter in the colon from a recent foraging bout.

### Statistical Analysis

Statistical analyses were performed using SPSS 12.0. Potential differences between receptive and non-receptive females were tested with an Independent Samples *t*-test. Male copulation success was investigated using an analysis of variance design (ANOVA) with display, fight, and chase rate as covariates. Correlations among receptive females were investigated with Pearson's product-moment correlations, and significance levels were adjusted to control for multiple comparisons using the false discovery rate procedure [40], which controls the expected proportion of null hypotheses that are falsely rejected. *P*-values are ranked in ascending order, and compared to a critical significance level, starting with the largest *P*-value. The critical significance level of each comparison, *d_i_*, is calculated by dividing the rank of the *P*-value by the total number of comparisons, and multiplying by the false discovery rate (0.05). The critical *P*-value, *P_k_*, is the first *P*-value, *P_i_*, that is less than or equal to *d_i_*. The null hypothesis is rejected for all comparisons with *P*-values≤*P_k_*. This method has greatly increased power over procedures that control the probability of rejecting any null hypotheses, such as Bonferroni corrections, particularly when dealing with small sample sizes [41], [42]. Effect sizes were calculated for all tests to provide a measure of the strength of the relationship between variables. Note that sample sizes differ in correlations because we were not always able to obtain measurements from each female on every factor. No available data was excluded from the analysis. All data are normally distributed and are expressed as means±s.e.m.

## References

[1] Milinski M, Bakker TC (1992). Costs influence sequential mate choice in sticklebacks, *Gasterosteus aculeatus*.. Proc R Soc Lond B.

[2] Kraak SBM, Weissing FJ (1996). Female preference for nests with many eggs: a cost-benefit analysis of female choice in fish with paternal care.. Behav Ecol.

[3] Gotthard K, Nylin S, Wiklund C (1999). Mating system evolution in response to search costs in the speckled wood butterfly, *Pararge aegeria*.. Behav Ecol Sociobiol.

[4] Luttbeg B, Towner MC, Wandesforde-Smith A, Mangel M, Foster SA (2001). State-dependent mate-assessment and mate-selection behavior in female threespine sticklebacks *(Gasterosteus aculeatus)*.. Ethology.

[5] Wong BBM, Jennions MD (2003). Costs influence male mate choice in a freshwater fish.. Proc R Soc Lond B.

[6] Reynolds JD, Gross MR (1992). Female mate preference enhances offspring growth and reproduction in a fish, *Poecilia reticulata*.. Proc R Soc Lond B.

[7] Norris K (1993). Heritable variation in a plumage indicator of viability in male great tits *Parus major*.. Nature.

[8] Petrie M (1994). Improved growth and survival of offspring of peacocks with more elaborate trains.. Nature.

[9] Tallamy DW, Darlington MB, Pesek JD, Powell BE (2003). Copulatory courtship signals male genetic quality in cucumber beetles.. Proc R Soc Lond B.

[10] Reynolds JD, Gross MR (1990). Costs and benefits of female mate choice: is there a lek paradox?. Am Nat.

[11] Kirkpatrick M (1996). Good genes and direct selection in evolution of mating preferences.. Evolution.

[12] Kirkpatrick M, Ryan M (1991). The evolution of mating preferences and the paradox of the lek.. Nature.

[13] Shuster SM, Wade MJ (2003). Mating systems and strategies..

[14] Borgia G, Blum MS, Blum NA (1979). Sexual selection and the evolution of mating systems.. Sexual Selection and Reproductive Competition in Insects..

[15] Pomiankowski A, Møller AP (1995). A resolution of the lek paradox.. Proc R Soc Lond B.

[16] Gibson RM, Bachman GC (1992). The costs of female choice in a lekking bird.. Behav Ecol.

[17] Byers JA, Wiseman PA, Jones L, Roffe TJ (2005). A large cost of female mate sampling in pronghorn.. Am Nat.

[18] van Marken Lichtenbelt WD, Wesselingh RA, Vogel JT, Albers KBM (1993). Energy budgets in free-living green iguanas in a seasonal environment.. Ecology.

[19] Wikelski M, Carbone C, Bednekoff PA, Choudhury S, Tebbich S (2001). Why is female choice not unanimous? Insights from costly mate sampling in marine iguanas.. Ethology.

[20] Rauch N (1985). Female habitat choice as a determinant of the reproductive success of the territorial male iguana *(Amblyrhynchus cristatus)*.. Behav Ecol Sociobiol.

[21] Wikelski M, Carbone C, Trillmich F (1996). Lekking in marine iguanas: female grouping and male reproductive strategies.. Anim Behav.

[22] Partecke J, von Haenseler A, Wikelski M (2001). Territory establishment patterns support the hotshot-hypothesis in lekking marine iguanas.. Behav Ecol Sociobiol.

[23] Trillmich KGK (1983). The mating system of the marine iguana *(Amblyrhynchus cristatus)*.. Z Tierpsychol.

[24] Butler PJ, Frappell PB, Wang T, Wikelski M (2002). The relationship between heart rate and rate of oxygen consumption in Galápagos marine iguanas *(Amblyrhynchus cristatus)* at two different temperatures.. J Exp Biol.

[25] Laurie WA (1990). Population biology of marine iguanas *(Amblyrhynchus cristatus)* I. Changes in fecundity related to a population crash.. J Anim Ecol.

[26] Hancock TV, Adolph SC, Gleeson TT (2001). Effect of activity duration on recovery and metabolic costs in the desert iguana *(Disposaurus dorsalis)*.. Comp Biochem Physiol.

[27] Laurie WA, Brown D (1990). Population biology of marine iguanas *(Amblyrhynchus cristatus)* II. Changes in annual survival rates and the effects of size, sex, age and fecundity in a population crash.. J Anim Ecol.

[28] Laurie WA, Brown D (1990). Population biology of marine iguanas (*Amblyrhynchus cristatus)* III. Factors affecting survival.. J Anim Ecol.

[29] in den Bosch HAJ (1998). Relationships between maternal size, egg size, clutch size, and hatchling size in European lacertid lizards.. J Herpetol.

[30] Angilletta MJ, Winters RS, Dunham AE (2000). Thermal effects on the energetics of lizard embryos: Implications for hatchling phenotypes.. Ecology.

[31] Wikelski M (2005). Evolution of body size in Galapagos marine iguanas.. Proc R Soc B.

[32] Miles DB, Fitzgerald LA, Snell HL (1995). Morphological correlates of locomotor performance in hatchling *Amblyrhynchus cristatus.*. Oecologia.

[33] Wikelski M, Carillo V, Trillmich F (1997). Energetic limits to body size in a grazing reptile, the Galapagos marine iguana.. Ecology.

[34] Head ML, Hunt J, Jennions MD, Brooks R (2005). The indirect benefits of mating with attractive males outweigh the direct costs.. PLoS Biol.

[35] Wikelski M, Romero LM (2003). Body size, performance and fitness in Galapagos marine iguanas.. Integr Comp Biol.

[36] Alatalo RV, Kotiaho J, Mappes J, Parri S (1998). Mate choice for offspring performance: major benefits or minor costs?. Proc R Soc B.

[37] Wikelski M, Hau M (1995). Is there an endogenous tidal foraging rhythm in marine iguanas?. J Biol Rhythms.

[38] Butler PJ, Green JA, Boyd IL, Speakman JR (2004). Measuring metabolic rate in the field: the pros and cons of the doubly labelled water and heart rate methods.. Funct Ecol.

[39] Gartrell BD, Girling JE, Edwards A, Jones SM (2002). Comparison of noninvasive methods for the evaluation of female reproductive condition in a large viviparous lizard, *Tiliqua nigrolutea*.. Zoo Biol.

[40] Benjamini Y, Hochberg Y (1995). Controlling the false discovery rate: a practical and powerful approach to multiple testing.. J R Statist Soc B.

[41] Nakagawa S (2004). A farewell to Bonferroni: the problems of low statistical power and publication bias.. Behav Ecol.

[42] Verhoeven KJF, Simonsen KL, McIntyre LM (2005). Implementing false discovery rate control: increasing your power.. Oikos.

